# Effects of Recreational Therapy and 3D Ultrasonography for High-Risk Pregnancies on Psychological Well-Being during Hospitalization and in the Puerperal Phase

**DOI:** 10.3390/jcm12196228

**Published:** 2023-09-27

**Authors:** Elna Kuehnle, Jessica Jungk, Lars Brodowski, Fabian Kohls, Peter Hillemanns, Ismini Staboulidou

**Affiliations:** Department of Obstetrics and Gynaecology, Hanover Medical School, Carl-Neuberg-Str. 1, 30625 Hannover, Germany

**Keywords:** high-risk pregnancies, hospitalization, recreational therapy, 3D ultrasonography, antenatal depression

## Abstract

Hospitalization during pregnancy often produces psychosocial distress for pregnant women. In this study, 3D ultrasound and recreational therapy were compared to the standard treatment for their influence on depressive symptoms and anxiety. In this prospective one-year intervention study, women who were admitted to the hospital for any pregnancy complication, other than psychiatric, were included. A control group, with standard clinical treatment, and two intervention groups, both additionally receiving either 3D ultrasound or recreational therapy, were established. Psychological well-being was assessed at defined times by the PHQ-health-questionnaire. A total of 169/211 women were included: control group *n* = 79, 3D ultrasound group *n* = 43, and crochet group *n* = 83. A higher than estimated underlying depression was seen for all women on admission. The intervention groups showed less depression (*p* = 0.02762). No difference was seen between the intervention groups (*p* = 0.23029). Anxiety decreased throughout intervention, but not significantly. On admission, all women showed similar results of underlying depression, indicating that hospitalization itself already causes mild psychological stress. Both interventions decreased depressive symptoms. Intervention with either recreational therapy or 3D ultrasound can prevent the development of mild and major depression and decrease anxiety disorders, and therefore has a positive effect on well-being during hospitalization. These results emphasize the need to implement forms of interventions to improve the well-being of women, as this might improve pregnancy and neonatal outcome.

## 1. Introduction

Pregnancy marks a special time in a woman’s life. On the one hand, there is the joy of becoming a mother and raising a child; on the other hand, there is also the pressure of the upcoming new responsibility for a child, together with the complete change this brings to one’s own life. Women experience a time of complete social, biological, and psychological change. Usually, women are well capable of managing the challenges of pregnancy, and the joy will predominate the concerns. Complications during pregnancy, such as hospitalization for different medical conditions in context with the pregnancy, can alter the balance, and can cause strong psychological stress in women [1,2,3].

Depressive and anxiety symptoms in healthy non-high-risk pregnancies are common complications after pregnancy, as well as during pregnancy. Although most studies focus on postnatal depression, there is a growing body of evidence that antenatal depression is a widespread complication during pregnancy [4,5,6]. In a meta-analysis of 21 studies, the mean prevalence rate of depression across the antennal period is 10.7% for a general pregnant population [7]. Other publications reported a prevalence for antenatal depression in 10–12%, 21%, 29.2%, and 37%, respectively [8,9,10,11]. Several risk factors for antenatal depression are currently discussed, for example, sociodemographic and medical risk factors [12]. 

This is not only of interest for the maternal well-being, but also for the course of pregnancy, as antenatal depression is known to play a significant and independent role in the prediction of preterm delivery [13,14]. The current literature describes an increased risk for miscarriage, preterm birth, preeclampsia, intrauterine growth restriction, and low birth weight infants in women with depression during pregnancy [15,16,17]. Antenatal depression is strongly associated with postnatal depressive symptoms, adverse perinatal outcomes [18], and a higher c-section rate [19]. If untreated, depression during pregnancy also increases the risk for postpartum depression, which has negative effects on maternal–infant attachment and child development [20].

Information on the frequency of antenatal anxiety in otherwise healthy pregnancies differs throughout the literature. Anderson et al. [21] described a prevalence of 6.6%. There is evidence for a concurrent relationship between anxiety in pregnancy and preterm birth [22], less optimal maternal–fetal quality of attachment, and more negative attitudes toward motherhood and the self-fulfilling prophecy postnatally [23]. Anxiety during pregnancy was found to be a significant predictor of mother–infant bonding disturbance in the postpartum period [24]. Prenatal attachment is the parent’s emotions, perceptions, and behaviors that are related to the fetus [25]. There is a relationship between the quality of prenatal parent–infant bonding and the quality of postnatal parent–infant relationship [26]. Therefore, it is important to identify factors that compromise prenatal parent–infant bonding, as well as factors helping to improve prenatal parent–infant bonding. The quality of the parent–infant relationship is an important factor influencing the child’s later well-being [25]. It is known that children who developed a secure relationship with their parents in their first years of life, generally have better cognitive outcomes and better social interactions [27]. Koutra K et al. [28] found evidence that antenatal and postnatal maternal psychological well-being has important consequences on early child neurodevelopment. Improving the mother’s prenatal well-being and parent–infant relationship, therefore, can lay the foundation stones for solid child development. 

Most studies as above-mentioned focus on healthy pregnant women in an out-patient setting, without obstetrics risk or need for hospitalization. High-risk pregnancies mostly are excluded from analysis. Hospitalization during pregnancy is known to increases maternal distress [29]. A prevalence between 28- 44% of depressive symptoms, or even major depression [11], is seen in cohorts hospitalized for obstetric complications [3,11,13].

As there is little information on the prevalence of depressive and anxiety symptoms for a high-risk pregnancy cohort during hospitalization, even fewer studies focus on the treatment of depressive and anxiety symptoms. Mostly, pharmacological or psychological interventions using cognitive behavioral therapy (CBT) are described [30,31,32,33]. Reduction of depressive symptoms and anxiety by using CBT is reported in different studies [34,35]. There is also growing evidence that behavioral therapy can reduce adverse pregnancy outcomes, e.g., preterm birth [14].

Other interventions, which are used to either prevent or treat antenatal depressive symptoms and anxiety, are reported. For example, music therapy [36] and recreation therapy, in general, have been described to improve maternal well-being during hospitalization [37,38]. A few studies also focus on the positive use of ultrasound to improve maternal well-being and maternal fetal bonding [39,40].

To the best of our knowledge, there is no study to date that has examined the prevalence of antenatal depression and anxiety during hospitalization for high-risk pregnancy, and the effect of crochet as a form of recreation therapy on maternal well-being. 

On the background of the known negative effects of hospitalization on pregnant women and the high prevalence of antenatal depression and anxiety and its known negative effect on pregnancy outcome and short- and long-term neonatal outcome, this study explored options for reducing the negative psychological effects of hospitalization by implementing recreational therapy or 3D ultrasound into everyday care during the hospital stay.

## 2. Materials and Methods

This longitudinal, prospective, interventional, single-center study was conducted at the Department of Obstetrics and Gynecology at the Hanover Medical School during a period of one year. All pregnant women, who were hospitalized for a minimum of 7 days or longer, were asked to participate in this study. In the case of written consent and compatibility with inclusion and exclusion criteria, they were included. This study was approved by the regional ethics committee of the Hanover Medical School, Nr. 2765-2015.

Inclusion criteria were hospitalization during pregnancy for any reason in context of the current pregnancy, for example, risk of preterm birth, fetal intrauterine growth restriction, or preeclampsia. 

Exclusion criteria were patients with either a history of psychiatric disorders under current therapy, or patients with an acute psychiatric disorder in need of acute psychiatric intervention. Additionally, patients under the age of 18 years were excluded from the study.

This was a longitudinal prospective interventional study, and patients were recruited according to a timeline. Each group was recruited for approximately 4 months after one after another, leading to different numbers of patients per group. First, a control group was built. They were treated according to the hospital standard, which is in accordance with German and international guidelines [41], depending on the reason for hospitalization. The second group received a weekly 3D ultrasound of the fetus, in addition to standard treatment by trained medical personnel. The third group was introduced to crochet, in addition to standard therapy. Crochet was introduced as a form of recreational therapy that is easy to learn, can be practiced at any time, and is in no conflict with bed rest. Women of this group were visited at least weekly by trained medical personnel to assist with crochet and provide new materials and ideas.

All women were given a standardized health questionnaire (short version of the PHQ-D Gesundheitsfragebogen für Patienten) [42] in order to assess their psychological status. The questionnaire was given on admission, after 1, 2, 4, 6, 8, 10, 12, weeks and in the postnatal period. 

This questionnaire includes 9 questions for the detection of depressive symptoms. Answers are categorized into four groups, with a point score for every answer. All 9 point scores are added and, depending on the value, the patients can be categorized into the following psychological status classification: no depression (0–4), underlying depression (5–9), and major depression (10–27). The group of major depression is additionally divided in 3 subgroups according to the severity of the major depression. The second part of the questionnaire includes five questions focusing on anxiety disorders. A yes answer indicates the existents of an anxiety disorder. The last part of the questionnaire looks into psychosocial functionality, and the patients are asked if the above-mentioned problems interfere with their everyday life. Four options are given in order to rate their ability to cope with everyday life.

The following two hypotheses were analyzed: (1) Women in both intervention groups have fewer depressive symptoms, less anxiety, and fewer problems coping with everyday life. (2) Women in the crochet group experience fewer depressive symptoms, fewer anxiety symptoms, and fewer problems coping with everyday life compared to the 3D ultrasound group.

Data from all included patients were collected using the patient’s hospital records. The statistical analysis was performed in collaboration with the Institute of Statistics of the Leibniz-University of Hanover, using the statistic program R Version 3.3.2 (http://www.cran.r-project.org accessed on February 2017). First, descriptive statistics were applied. Second, a panel regression model was used. The same questionnaire was used at defined points of time on the same patients, but the number of questioned patients decreased over time due to hospital discharge, meaning an unbalanced panel exists. Statistical significance is achieved if *p* < 0.05.

## 3. Results

A total of 211 patients participated in this study. A total of 42 patients were discharged from the hospital within less than 7 days. A total of 169 stayed for at least one week. The control group (Group 1) consisted of 79 patients, the ultrasound group (Group 2) of 49 patients, and the crochet group (Group 3) of 83 patients. 

A couple of reasons lead to fewer patients receiving interventions in Groups 2 and 3. In Group 2, 31 (61.3%) patients received 3D ultrasound, and in Group 3, 32 (49.2%) patients did crochet. 

See Table 1 for patient characteristics.

Overall, our study population presented with major depression in 21.80%, and mild depression in 43.60% on admittance to hospital. Symptoms of anxiety were seen in 5.67% of the whole study population.

**Figure 1:**
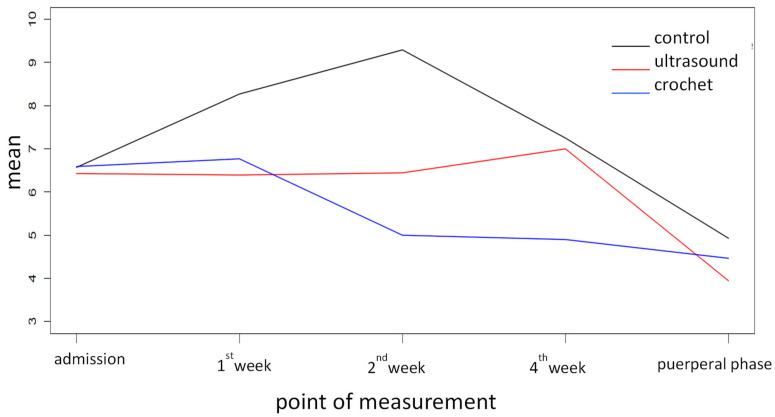
Mean point score of depressive symptoms for all tested groups over time.

First, the mean point score for depressive symptoms was determined on all measuring points for Group 1. The highest points, with 9.29 points, indicating a mild depression, were seen at week 2, while the lowest were seen during the puerperal phase, with 4.93, indicating no depression. The mean point score for Group 2, with 8 points, was detected in week 4, and the lowest in the puerperal phase, with 3.94. Compared to the control group, there is a lesser variation. Group 3 showed the highest number of points after the first week, with 6.77 points, indicating mild depressive symptoms, and the lowest points after 8 weeks. Overall, we see the least variation between means in Group 3. Comparing all three groups, nearly similar scores are seen at the time of admission and in the puerperal phase. At week 2, the greatest differences are seen between the three groups. While the control group reaches the highest score, with 9.29, close to the categorization of a major depression, the crochet group has the lowest score, with 5.00, close to the categorization of no depression. Group 2 ranks in-between, with 6.44. See Figure 1.

In this study, the same women were questioned with the same questionnaire at different specific times. The analysis was conducted for the following points of time: admission to hospital, after 1, 2, and 4 weeks of hospitalization, and during the puerperal phase. For this analysis, not all above mentioned points of time were considered, due to a decreasing number of patients in all groups with ongoing duration of hospital stay. The influence of the following variables was investigated: intervention, duration of hospitalization, pregnancy week, parity, school education, bedrest, tocolysis, and age.

First, hypothesis 1 was tested. To assess the influence of intervention in the form of recreational therapy or 3D ultrasonography on psychological well-being, Groups 2 and 3 were compared with the control group (Group 1). After adding all variables to the panel regression model, and then using the general-to-specific model selection to erase all insignificant variables, only week of pregnancy and intervention vs. standard therapy were significant variables. The intervention groups (3D ultrasound and crochet) showed fewer depressive symptoms compared to standard treatment (Group 1) (*p* = 0.02762). Additionally, women in a higher pregnancy week showed significantly fewer depressive symptoms (*p* = 0.01118). Bedrest did increase depressive symptoms, but this was not statistically significant (*p* = 0.58). When only the classification model for depression is used, intervention is not a significant variable, no significant difference is seen between control and intervention groups (*p* = 0.2167).

Tested for anxiety, bedrest (*p* = 0.000497), parity (*p* = 0.002989), and pregnancy week (*p* = 0.020897) significantly increased the degree of anxiety in the control group compared to the intervention groups.

A higher pregnancy week decreased difficulties in everyday life (*p* = 0.000000) of both intervention groups, while age increased difficulties in everyday life (*p* = 0.000979) compared to the control group.

Second, hypothesis 2 was tested. The intervention groups were compared to each other in order to investigate a possible influence of the form of intervention. Group 2 (ultrasound) was compared with Group 3 (crochet), again using all the variables for the above-mentioned endpoints. Belonging to one of the two groups did not have a significant difference on the influence on depressive symptoms, neither using points score nor using the classification system (*p* = 0.23029). Using the points score, and after adjusting with the general-to-specific model, only a higher pregnancy week (*p* = 0.02559) and given birth before (*p* = 0.02858) decreased depression in the crochet group, while a higher age (*p* = 0.01601) increased depression. Applying the classification model, no significant influence of any variable was seen. 

After analysis, only bedrest (*p* = 0.0077) and higher age (*p* = 0.0147) led to a higher degree of anxiety. No significant differences between the two groups were seen. Coping with everyday life was compromised by higher age (*p* = 0.0000), while a higher pregnancy week significantly decreased difficulties with everyday life (*p* = 0.0000). Again, the form of intervention did not influence coping with everyday life.

## 4. Discussion

This study examined the prevalence of depressive and anxiety symptoms in a group of pregnant women that are at even higher risk of developing symptoms of depression and anxiety, and feasible prevention and treatment options. Overall, major depression was seen in 21.80%, and mild depression in 43.60% of all patients on admittance to hospital, indicated by the PHQ questionnaire in this study population. This is in concordance with results from another study [11] for a high-risk hospitalized pregnant population. The rate of antenatal anxiety is inconclusive throughout the current literature. In this study we found a prevalence of 5.67%. Although anxiety during pregnancy is less common compared to depression, it should receive close attention and treatment for its known negative effects on fetal–parental bonding, child development, and cognitive behavior [24,25,27]. 

The high incidence of an underlying depression, which would not have been identified outside of this study, in parts of a hospitalized pregnant women population should alert each clinician to be aware of this possibility, and screening tools should be implemented into clinical routine. 

Hospitalization during pregnancy that was not for labor was seen in 13% of all pregnancies in Germany over several years [43], and 28.17% of these with a duration of 7 days or longer. This indicates the relevance of hospitalization during pregnancy, and the need to further investigate its effects. 

Women with a prior history of mental illness are at higher risk to develop antenatal depression [44]. As we excluded all women with a history of mental illness or in need of acute psychiatric help, the results of this study are a resemblance of the stressful influence of hospitalization itself during pregnancy on pregnant women, as we did see underlying major depression in 21.8% of the whole study population at the beginning of hospitalization. This is well above the assessed average depression seen in healthy nonhospitalized pregnant women in other studies [6,9]. Interestingly, in our study, the course of depressive symptoms decreases in all groups closer to the end of the pregnancy, and with the lowest score in the postpartum period. One can argue that women feel more secure, as with advancing pregnancy the risk for preterm delivery and the risk of anticipated neonatal complications decrease. These are not concerns anymore in the postpartum period, where the stressor of hospitalization is not apparent anymore. 

Bedrest, age, parity, and no intervention vs. intervention were all factors negatively affecting depressive symptoms and anxiety in our cohort. Bedrest is still a commonly used therapy during hospitalization for threatening preterm labor, although the current literature does not find any evidence of improvement [45]. Instead, more negative effects of bedrest on general health, as well as pregnancy outcome, are seen [46,47,48]. In this study, bedrest increased depressive symptoms and anxiety, and is another strong argument to leave bedrest as a medical treatment behind.

Parity is discussed with controversy throughout the literature, for either being a risk factor for depression [49,50] or having no association [21,51]. Our study found no influence of parity on depressive symptoms, but anxiety was increased by parity. Teixeira reported a significant interaction between parity and trimester of pregnancy for anxiety symptoms. There was higher anxiety in the first trimester of primiparous women compared with the 3rd trimester, and vice versa with multiparous. Having to raise another child seems to be a stressor [49,51].

Additionally, age correlates with more anxiety and depression [23]. This is especially interesting because of the increasing rates of older ages in women having their first-born child in Germany [52]. We did detect a correlation between age and anxiety/depression.

This study detected a positive influence of intervention either by ultrasound or by crochet on depressive symptoms. There is an ongoing controversial discussion on the use of ultrasonography during pregnancy, for other than diagnostic reasons, that we are aware of. This study used 3D ultrasonography to improve maternal well-being through improvement of maternal–fetal attachment in a high-risk pregnancy collective. Therefore, the general discussion does not apply for our collective. Still though, the use of 3D ultrasonography should be limited to diagnostic and medical reasons, or in clinical studies.

In a systematic review and meta-analysis, Fontain-Kuipers et al. [53] compared the effect of different interventions to reduce maternal distress during pregnancy. These interventions were mostly psychological methods, rather than recreational practical interventions. There was no significant positive effect of all interventions on the general pregnant population, except on women with a predisposition or already suffering from maternal distress. This is in concordance with the results of this study. Hospitalization and high-risk pregnancy are stressors, and lead to an increase in depressive symptoms, as well as a high anxiety rate. Interventions such as crochet or 3D ultrasonography are able to alleviate symptoms. Both methods showed feasibility in clinical routine, although they mean an increased personnel effort. This will likely limit their use, especially with the restricted financial and personnel resources the health care system faces today. On the other hand, one should consider the high social and financial burden of preterm birth, and the long-term effects caused by adverse obstetric and neonatal outcomes.

There are limitations to this study due to methodology and sample size. As this is a single-center study in a level one university hospital, the prevalence of severe pregnancy complications in the cohort is well above average. Therefore, generalization of prevalence of depression and anxiety symptoms on all hospitalized pregnant women should not be made. 

This being a single-center pilot intervention study, sample size and balance between groups are limited. The voluntary nature causes bias, especially in the intervention groups. Not all women found themselves eligible for crochet, and dropped out. 

We used the health questionnaire for patients (PHQ-D Gesundheitsfragebogen für Patienten) [42] for the detection of depression, anxiety, and coping with everyday life. In the current literature, there are several different tests used to screen for depression and anxiety. A widely used and validated psychological test is the EPDS. This test is especially designed for postnatal depression, but is increasingly used to asses antenatal depression as well. For example, Andersson et al., Hermon N et al., and Eastwood J at al. [6,13,18] used this test for antenatal depression, whereas others such as Koutra K et al. [28] combined it with the STAI trait for anxiety symptoms. Although the EPDS is a commonly used instrument, it has its limitations due to length and interpretation. Most studies only questioned the patients once, making the EPDS a good instrument. As we planned a sequential interview mode, the EPDS seemed too long, and the compliance would have decreased. Therefore, we chose the PHQ questionnaire, as it is a short questionnaire limited to one page. There is an 83–87% match for the evaluation of depression between these two tests [54,55]. Still, this marks a limitation to our study, making results less comparable to other studies. For the detection of anxiety, most other studies used the state-trait-anxiety-inventory (STAI). This questionnaire is much longer than the PHQ-D, making it less favorable for this study concept. With a reply rate as high as 85% overall, the PHQ-D showed itself feasible for this study design. Additionally, our study is the first, to our knowledge, that assessed the development of depression, anxiety, and coping with everyday life over a period of time during pregnancy in a hospital setting and in the postnatal phase, making this study more solid for long-term evaluation.

## 5. Conclusions

In summary this study found a surprisingly high prevalence of depressive symptoms and anxiety in a hospitalized high-risk pregnancy population. Interventions such as recreational therapy or 3D ultrasound are equally capable of alleviating these symptoms, and therefore might be effective methods to improve pregnancy and fetal outcome.

In the future, studies should explore short- and long-term fetal outcome after treatment of antenatal depression with different interventions. Feasible and effective low-threshold interventions need to be identified, tested for efficacy, and implemented in clinical routine.

## Figures and Tables

**Table 1:** Patient characteristics.

	All	Group 1	Group 2	Group 3
Number of Patients	211	79	49	83
Mean	n (%)	n (%)	n (%)	n (%)
Age (years)	32	32	32	31
Hight (cm)	166	167	165	166
Weight (kg)	70	70	68	71
Week of pregnancy on admittance	29	28	28	29
Duration of hospital stay (days)	18	21	16	17
Parity				
Primipara	111 (52.6)	45 (57.0)	25 (51.0)	41 (49.4)
Multipara	100 (47.4)	34 (43.0)	24 (49.0)	42 (50.6)
Gravidity				
Primigravida	86 (40.8)	33 (41.8)	18 (36.7)	35 (42.2)
Multigravida	125 (59.2)	46 (58.2)	31 (63.3)	48 (57.8)
Singelton pregnancy	165 (78.2)	54 (68.4)	41 (83.7)	70 (84.3)
Twins or more	46 (21.8)	25 (31.6)	8 (16.3)	13 (15.7)
Alcohol consumption	5 (2.4)	0 (0.0)	2 (4.1)	3 (3.6)
Smoking	40 (19.0)	13 (16.5)	13 (26.5)	14 (16.9)
Therapy				
Bedrest	139 (65.9)	51 (64.6)	35 (71.4)	53 (63.9)
Tocolysis	57 (27.0)	17 (21.5)	14 (28.6)	26 (31.3)

## Data Availability

Data can be acquired from author directly. Contact: kuehnle.elna@mh-hannover.de.
